# Airway Smooth Muscle Dynamics and Hyperresponsiveness: In and outside the Clinic

**DOI:** 10.1155/2012/157047

**Published:** 2012-10-17

**Authors:** Peter B. Noble, Thomas K. Ansell, Alan L. James, Peter K. McFawn, Howard W. Mitchell

**Affiliations:** ^1^Centre for Neonatal Research and Education, School of Women's and Infants' Health, The University of Western Australia, Crawley 6009, Australia; ^2^School of Anatomy, Physiology, and Human Biology, The University of Western Australia, Crawley 6009, Australia; ^3^Department of Pulmonary Physiology, West Australian Sleep Disorders Research Institute, Sir Charles Gairdner Hospital, Nedlands 6009, Australia; ^4^School of Medicine and Pharmacology, The University of Western Australia, Crawley 6009, Australia

## Abstract

The primary functional abnormality in asthma is airway hyperresponsiveness (AHR)—excessive airway narrowing to bronchoconstrictor stimuli. Our understanding of the underlying mechanism(s) producing AHR is incomplete. While structure-function relationships have been evoked to explain AHR (e.g., increased airway smooth muscle (ASM) mass in asthma) more recently there has been a focus on how the dynamic mechanical environment of the lung impacts airway responsiveness in health and disease. The effects of breathing movements such as deep inspiration reveal innate protective mechanisms in healthy individuals that are likely mediated by dynamic ASM stretch but which may be impaired in asthmatic patients and thereby facilitate AHR. This perspective considers the evidence for and against a role of dynamic ASM stretch in limiting the capacity of airways to narrow excessively. We propose that lung function measured after bronchial provocation in the laboratory and changes in lung function perceived by the patient in everyday life may be quite different in their dependence on dynamic ASM stretch.

## 1. Introduction

Excessive and variable airway narrowing is the primary functional impairment observed in patients diagnosed with asthma—usually on a basis of variable wheeze, shortness of breath, cough, and chest tightness that responds well to bronchodilators. In the laboratory this is associated with apparent increased sensitivity (left-ward shift in the dose-response curve) and maximal response to inhaled bronchoconstricting agents [1]. These functional abnormalities are collectively referred to as airway hyperresponsiveness (AHR). In patients with asthma, AHR predicts the susceptibility for an increased rate of decline in lung function [2], increased risk of exacerbations and increased requirements for inhaled corticosteroids [3, 4]. Identifying the mechanism(s) producing AHR in asthma has been a priority research focus over many decades, but our understanding of the pathophysiology of asthma remains incomplete. Explanations for AHR which focussed on the “static” structure-function models of excessive airway narrowing [5, 6] have more recently incorporated the integrated dynamic properties of airway smooth muscle (ASM) [7, 8]. This perspective considers the evidence for and against a role of dynamic ASM stretch in limiting the capacity of airways to narrow excessively, failure of which is proposed as a cause of AHR [9]. Other mechanisms relating dynamic ASM stretch to altered airway calibre include neural, hormonal, and paracrine pathways. These have been summarised previously [10] and will not be discussed in this paper.

## 2. *In Vivo* Response to Deep Inspiration: Establishing the Hypothesis

The importance of lung volume to airway responsiveness is well recognised: a small increase in volume produces a substantial reduction in bronchoconstriction [11]. Similarly, studies that alter positive end expiratory pressure (PEEP) also report a strong inhibitory effect of lung volume on bronchoconstriction [12–14]. The role of the dynamic volumes during breathing, as distinct from persist changes in volume occurring with PEEP, is demonstrated *in vivo* by observing how a deep inspiration (DI) transiently stretches the airway wall. Skloot et al. [15] showed that when healthy subjects avoid taking DIs during bronchial challenge, the resulting dose-response curves resembles those of asthmatic patients. That is, the exclusion of DIs from the normal breathing rhythm was seen to increase bronchoconstriction. These findings, combined with observations that the respiratory response to DI is reduced or absent in asthmatic subjects [16–18], and in some patients DI even augments bronchoconstriction [19], suggest that dynamic stretch is an important determinant of airway responsiveness and an abnormality in this protective mechanism could facilitate AHR. However an impaired response to DI in asthma may not account for all features of AHR such as increased sensitivity. The sensitivity of bronchoconstrictor response is not influenced by the presence of DI during provocation challenge [20, 21]. 

The benefits of DI in healthy individuals include reversal of existing bronchoconstriction (bronchodilation) [20, 22, 23] and attenuation of bronchoconstriction induced following DI (bronchoprotection) [24–26], both of which are reduced in asthmatic individuals [20, 25–27]. The underlying mechanisms of bronchodilation and bronchoprotection may or may not be distinct [24]. The apparent bronchoprotective effects of DI, undertaken prior to bronchial challenge, could involve an enhanced bronchodilatory response since the degree of constriction is measured by the FEV_1_ which itself is preceded by a DI and likely to produce bronchodilation [27–29]. Compared with DI, the separate effects of tidal breathing are more difficult to assess, but have been explored in mechanically ventilated animals and are also effective in limiting bronchoconstriction [30–32]. 

## 3. Evidence from Isolated ASM

In isolated tracheal ASM strips, the effects of dynamic breathing movements have been simulated by length oscillations prior to or during activation of the ASM, typically by exogenous muscarinic agents or parasympathetic nerve stimulation [8, 33–38]. Length oscillation during ASM activation resulted in a marked decrease in ASM force production [8, 33, 38] and shortening [34], in proportion to the amplitude of length oscillation. Importantly, it is proposed [8] that large changes in ASM force will occur to length oscillation accompanying tidal breathing (estimated from lung volumes and assuming isotropic expansion). Cellular mechanisms include cross-bridge detachment due to lengthening, the so-called “perturbed equilibrium hypothesis” [34, 39]. 

Length oscillation of ASM prior to activation is also effective in modulating ASM force and this is similarly dependent on the amplitude of length change [35–37]. Plasticity of ASM force-length properties (length adaptation) has been evoked to explain the effect of length change on the relaxed cell via remodelling of the contractile apparatus [40–42]. Length adaptation or plasticity at least theoretically explains both the bronchodilatory and bronchoprotective effects of DI [43, 44]. 

## 4. Evidence from Bronchial Segments

Whole bronchial segments that retain the normal architecture of ASM and connections with other mural components have been used to study the effect of dynamic stretch on ASM contraction. Gunst et al. [45] applied fixed volume oscillations to canine airway segments and examined the effects of bronchoconstriction to acetylcholine. They showed a pronounced reduction in the contractile response (narrowing and pressure generation) during volume oscillation, findings qualitatively similar to those in isolated ASM *in vitro* [8, 33, 38] and in mechanical ventilated animals *in vivo* [30–32]. Subsequently, numerous other studies using porcine airway segments confirmed that volume oscillations suppress bronchoconstriction [46–48]. However, what is clear is that pressures accompanying volume oscillation in airway segments become very large during contractile activation, a function of ASM stiffening [49].

**Figure 1:**
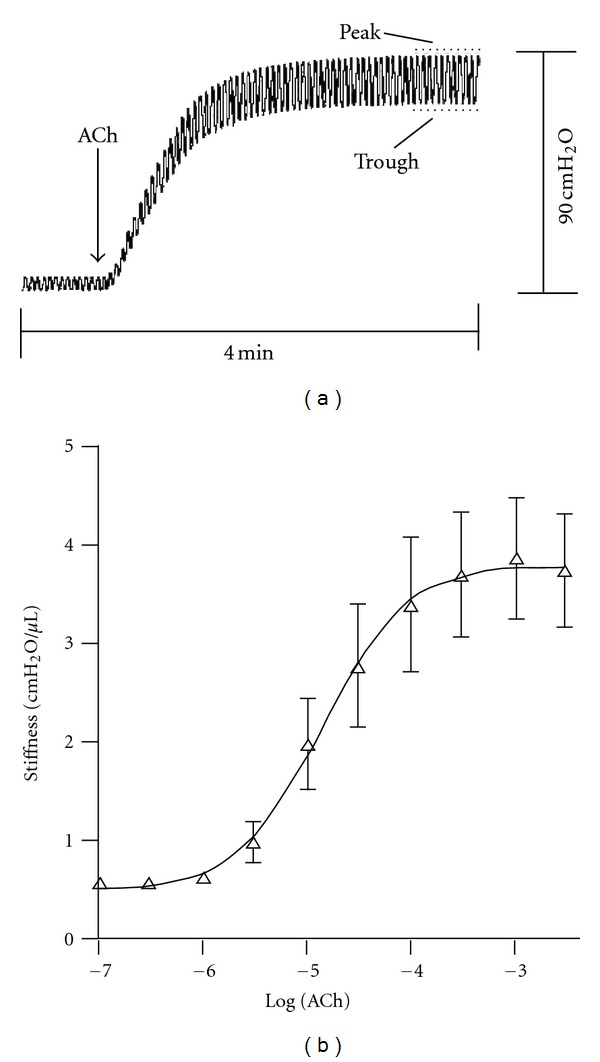
From [48]. (a) Lumen pressure fluctuations in isolated bronchial segments (porcine) during tidal volume oscillation before and after a maximal dose of acetylcholine (ACh). Tidal volume oscillations produced a trough-to-peak pressure cycle from 5 to 10 cmH_2_O in relaxed airways. Contraction to ACh is seen by the elevation in trough pressure in a closed system. The increase in the amplitude of pressure cycles indicates stiffening of the airway wall to ACh. (b) Sigmoidal dose-response behaviour of ACh-induced increase in airway stiffness. Values are means ± SE (*n* = 5).

We found that although baseline transmural pressures and volumes were chosen to simulate tidal breathing in the relaxed airway (i.e., Δ*P* = 5 cmH_2_O), the pressure swings associated with fixed volume oscillation during ASM stimulation increased ~four fold (Figure 1). When volume oscillations that produced more physiological pressure swings were used during ASM activation the effect of oscillation was greatly attenuated [48]. These observations lead us to conclude that the effects of dynamic stretch are limited by wall stiffness and that tidal oscillations are unlikely to significantly impact airway responsiveness. This conclusion was supported by LaPrad et al. [50] who applied fixed pressure oscillations on bovine airway segments and measured the effect on airway narrowing measured by ultrasound imaging. Under fixed pressure conditions airway narrowing was unaffected by tidal oscillations, casting doubt on the role of tidal oscillations in determining airway responsiveness [51].

Bronchial segment studies have also examined the effect of short-term inflations simulating DI, typically defined as inflation to 30 cmH_2_O which corresponds to transpulmonary pressure at the plateau of the lung pressure volume relationship [52]. In porcine airway segments DI produces potent, transient bronchodilation, largely dissipating within ~1 min [53, 54]. The magnitude and time-course of the bronchodilatory response in airway segments are consistent with bronchodilation to DI observed *in vivo* [22] suggesting that the airway wall response to dynamic stretch mediates this effect. Bronchodilatory responses to DI in whole airways are inversely proportional to airway wall stiffness and proportional to the magnitude of ASM stretch [48].

The level of ASM activation induced *in vitro* clearly impacts on the response to dynamic respiratory manoeuvres. Notably, bronchodilatory responses to DI in airway segments are observed under submaximal narrowing conditions (~30–40% decrease in lumen area) [53, 54] which should still be sufficient to produce large reductions in flow (~50–60% assuming homogenous constriction and laminar flow). However bronchodilatory responses to DI become diminished with increasing levels of ASM activation [48], and conversely, fixed pressure tidal oscillations can be effective under levels of activation at the bottom of the *in vitro* dose-response curve [53]. A question thus arises whether examining the response to dynamic mechanical stretch at maximal or near maximal levels *in vitro* is more relevant to disease, that is, asthma. 

The animal models used in studies utilising airway segments and muscle strips introduce the question of possible species differences in the role of dynamic ASM strain [55]. There are some differences between species that could impact the response to tidal or DI breathing, for example the porcine airway has a more abundant cartilaginous wall than the human airway which increases stiffness [56], while the bovine airway exhibits a myogenic response to simulated DI [50, 57] seemingly more in line with the bronchoconstrictor response after DI observed in some asthmatic patients [19]. Translational studies using human tissue are therefore necessary to confirm or extend findings in animal models. As discussed below, broadly speaking there is good agreement between studies utilising human tissue with those working with animal models. 

## 5. Human Tissue

To our knowledge four studies have reported the responses of human ASM to dynamic stretch. Tracheal ASM from nonasthmatic nontransplantable human lungs [58] showed attenuated force production following length oscillation, confirming findings from animal models. The same group also reported that in subjects with asthma the protective response to length oscillation was partially impaired [59]. This suggests that the reduced response to DI in asthmatic subjects may result from an impaired response of the ASM to mechanical stretch.

**Figure 2:**
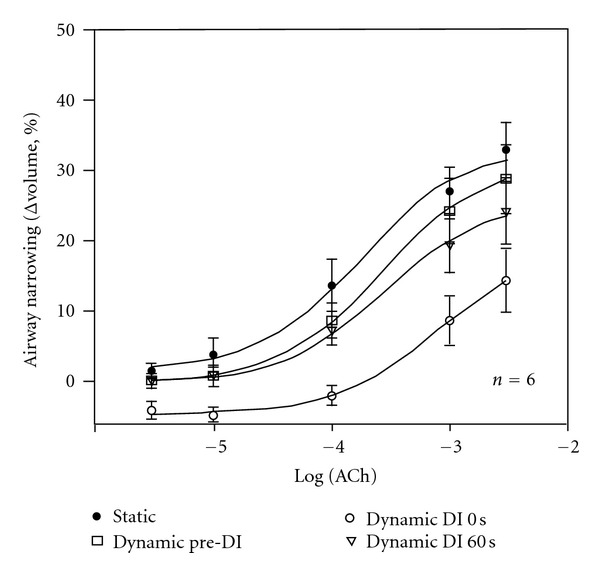
From [60]. Airway narrowing (Δvolume, %) to acetylcholine (ACh) in human bronchial segments. ACh dose-response curves were constructed from measurements of airway narrowing under static conditions (Static, 5 cmH_2_O) and during fixed transmural pressure cycles simulating tidal (5 to 10 cmH_2_O at 0.25 Hz) and deep inspiration (DI, 5 to 30 cmH_2_O). The dynamic pre-DI curve represents airway narrowing before the onset of DI; dynamic DI 0 s, the airway narrowing measured immediately after DI; dynamic DI 60 s, the airway narrowing measured 1 min after DI. DI produced an immediate reduction in maximal airway narrowing (*P* < 0.001) but not sensitivity. The effects of DI were largely ablated after 1 min. Airway narrowing under static conditions was not different to that prior to DI, suggesting that tidal oscillations alone did not regulate airway narrowing. Values are means ± SE (*n* = 6).

We examined the effects of simulated breathing manoeuvres in human bronchial segments [60] using tidal oscillations and DIs that mimicked the fixed pressure swing protocols described above [50, 53, 54]. Airway narrowing in tidally oscillated airways was reversed immediately after DI (Figure 2), followed by reconstriction over the course of 1 min. While the study did not examine the independent effects of tidal oscillation, the level of airway narrowing before the initiation of DI was similar to that under static conditions, arguing against an effect of tidal oscillation on airway narrowing. 

A human lung slice model has been used recently to examine the effect of tidal and deep breathing on airway narrowing [61]. A constant stress perturbation was applied to the lung slice, thereby imparting strain to the airway wall. The major results of the study confirm many of the previous findings from both animal and human tissue. Airway narrowing was reversed by deep “breathing” but not smaller “breaths.” In particular, tidal oscillations were ineffective. The effectiveness of breathing to antagonise airway narrowing increased with the level of wall stretch and decreased with the greater levels of contractile activation and wall stiffening.

## 6. ASM Dynamics and AHR in the Lung ****Function Laboratory

Many of the effects of DI are reasonably explained by the responses to dynamic stretch observed in isolated ASM and airway tissue *in vitro* and these will impact on measurements of airway responsiveness in the clinic. That is, since the traditional measure of airway responses to bronchoconstrictor agents is the FEV_1_, the DI which precedes the forced expiratory manoeuvre will produce bronchodilation and a differential response to DI between healthy and asthmatic subjects will result in a divergence of the dose-response curves [20]. Certainly, bronchodilator responses to DI are transient [17, 22], but in the context of a conventional bronchial challenge any bronchodilation, no matter how short-lived, will influence the FEV_1_ parameter. Extrapolating from the behaviour of individual airways [60], the magnitude of this effect approaches a halving in maximal response (Figure 2).

The mechanism producing AHR is however more than just an abnormal response to DI. Abnormal bronchodilatory responses to DI cannot explain AHR when constrictor responses are measured without the need for a DI (as required for FEV_1_) such as the forced oscillation technique [62]. As discussed, the effects of DI also do not explain any increased sensitivity of response in asthma since the position of the dose-response curve is not altered by the presence of DI during bronchial challenge [20, 21]. Some authors argue that the effect of DI in regulating the maximal response to bronchoconstrictor challenge in the clinical laboratory may *“artificially enhance the differences in responsiveness between healthy and asthmatic subjects”* [51]. Outside the boundaries set within the lung function laboratory, and putting FEV_1_ aside which provides just a snapshot of airway function, the dependence of lung function on DI as perceived by the patient may be quite different. 

## 7. ASM Dynamics and AHR outside the Lung Function Laboratory

Debate remains regarding whether airway responsiveness (the capacity for airways to narrow and restrict airflow) is suppressed by tidal oscillations and regular deep breaths, occurring in the form of spontaneous sighs at a rate of one in every six minutes [63]. The bronchodilatory responses to DI will of course have some effect but whether such transient bronchodilator events, which as discussed are influential in the measurement of the clinically derived FEV_1_ parameter, are frequent enough to be of major consequence to a patient in everyday life is uncertain. With respect to tidal oscillation, the evidence is mounting that under conditions where pressure fluctuations across the airway wall are constant (the scenario which is expected to occur with tidal breathing *in vivo*) these perturbations will have little to no effect on airway narrowing [48, 50, 60, 61].

The above leads to a conclusion that in the context of set limits of stiffness and strain the dynamic environment plays an important role in the measurement of airway responsiveness, as performed in the laboratory. But, given the kinetics of the dynamic response, perhaps it is unlikely to play a role in the day-to-day symptoms of the patient, that is, feelings of wheeze or chest tightness experienced by asthmatic individuals.

We need to try and resolve the apparent discrepancies between findings *in vivo* and *in vitro*. Tidal oscillations in mechanically ventilated animals *in vivo* demonstrate physiologically meaningful effects on airway narrowing [30–32] which is inconsistent with studies in isolated airways and lung slices *in vitro* [48, 50, 60, 61]. Interestingly, it was only since mechanical “limits” (i.e., pressure) were superimposed on our biological models that the effects of dynamic stretch appeared less effective [48]. Do pressure oscillations remain fixed during contractile activation *in vivo*? In studies on mechanically ventilated animals, it is tidal *volume* rather than pressure that is held fixed and this may account for the greater potency of tidal oscillations in this scenario. As the impedance of the system is increased with bronchoconstrictor challenge, respiratory pressures would be expected to increase for a constant volume change as observed in mechanically ventilated dogs [31]. Alternative explanations have also been proposed including an elevation in mean airway pressure during mechanical ventilation [50], however, this possibility has been empirically tested and only partially explains beneficial responses to tidal volume oscillations [31]. 

If we then consider what happens when a patient undergoes bronchoconstriction, in order to maintain adequate minute ventilation respiratory pressures may also increase to overcome the greater system impedance, although this will be influenced by the magnitude of the bronchoconstrictor response. Perhaps the true physiological simulation is one that exists somewhere between the fixed volume and pressure protocols previously described. The true biological effect of tidal oscillation then exists somewhere between these limits and may be greater than what has been suggested in recent studies [48, 50, 60, 61]. Indeed regular deep breathing is effective in reversing induced bronchoconstriction [64]. 

A final consideration is how bronchoprotective effects of DI influence bronchoconstriction *in vivo*. Unlike isolated ASM which exhibits a bronchoprotective-like effect whereby prior mechanical stretch reduces ASM force [35–37], bronchoprotection is not observed in midsized whole airways *in vitro* [60, 65]. The response of the whole airway is consistent with global *in vivo* measures of airflow and resistance that reveal no protective effect of prior DI on airway narrowing [27, 28, 66, 67]. The bronchoprotective effects of DI instead reduce the tendency towards airway closure, possibly by reducing airway surface tension [66]. The mechanism of DI-induced bronchoprotection may therefore involve more than an effect of mechanical stretch on the ASM.

## 8. Beyond ASM Dynamics

The role of ASM dynamics in the development of AHR should not be considered in isolation from other likely mechanism(s) including the effect of a thickened ASM layer in asthma [68]. The most intuitive explanation for an increase in maximal airway narrowing is enhanced ASM force due to greater ASM mass. This possibility is supported by mathematical simulations [5] but still lacks confirmatory biological data. The importance of ASM mass to AHR was well demonstrated using a murine gene knockout model of early growth response-1 which following stimulation with transforming growth factor alpha has pronounced ASM thickening and a severe form of AHR (compared with other models) [69]. The ASM growth was attributed solely to ASM hyperplasia which is the predominant pathology seen in severe asthma [70]. 

Neither increased ASM mass nor altered ASM dynamics account for changes in airway sensitivity. On reflection this is not surprising given the fact that mechanisms controlling sensitivity and maximal response (of the ASM and intact airways) differ [6]. The role of the epithelial mechanical barrier in limiting sensitivity to bronchoconstrictor stimuli was demonstrated decades ago by use of whole bronchial airway models *in vitro* [71, 72], similar to those described elsewhere in this paper. In intact airways the accessibility of ASM to agents applied to the airway lumen provides one of the strongest regulators of sensitivity. The original studies have been revisited recently in a mouse model [73].

## 9. Concluding Statements

The evidence from studies examining isolated ASM and whole airway behaviour *in vitro* suggests that dynamic ASM stretch is one determinant of airway responsiveness. The magnitude of this effect is dependent on the limits of these biological models including the magnitude of airway stretch, stress and ASM activation. It is unclear whether the effects of dynamic ASM stretch observed in the context of lung function measurements also influence the clinical symptoms of asthma. However, dynamic ASM stretch is unlikely to be the sole determinant of airway responsiveness and any impairment of this regulatory mechanism will interact with other pathological changes to produce AHR.
